# Distinct SARS-CoV-2 specific NLRP3 and IL-1β responses in T cells of aging patients during acute COVID-19 infection

**DOI:** 10.3389/fimmu.2023.1231087

**Published:** 2023-09-18

**Authors:** Shanmuga Sundaram Mahalingam, Sangeetha Jayaraman, Adhvika Arunkumar, Holly M. Dudley, Donald D. Anthony, Carey L. Shive, Jeffrey M. Jacobson, Pushpa Pandiyan

**Affiliations:** ^1^Department of Biological Sciences, School of Dental Medicine, Case Western Reserve University, Cleveland, OH, United States; ^2^Department of Molecular Biology and Microbiology, School of Medicine, Case Western Reserve University, Cleveland, OH, United States; ^3^Department of Rheumatology, Louis Stokes Cleveland Veterans Affairs Medical Center, Cleveland, OH, United States; ^4^Department of Pathology, Case Western Reserve University, Cleveland, OH, United States; ^5^Center for AIDS Research, School of Medicine, Case Western Reserve University, Cleveland, OH, United States; ^6^Department of Medicine, School of Medicine, University Hospitals, Case Western Reserve University, Cleveland, OH, United States

**Keywords:** aging, IL-1b, CD8 IFNγ cells + +, CD4, IFN-γ, immune senescence, COVID

## Abstract

Severe acute respiratory syndrome coronavirus 2 (SARS-CoV-2) causes Coronavirus Disease 2019 (COVID-19) that presents with varied clinical manifestations ranging from asymptomatic or mild infections and pneumonia to severe cases associated with cytokine storm, acute respiratory distress syndrome (ARDS), and even death. The underlying mechanisms contributing to these differences are unclear, although exacerbated inflammatory sequelae resulting from infection have been implicated. While advanced aging is a known risk factor, the precise immune parameters that determine the outcome of SARS-CoV-2 infection in elderly individuals are not understood. Here, we found aging-associated (age ≥61) intrinsic changes in T cell responses when compared to those from individuals aged ≤ 60, even among COVID-positive patients with mild symptoms. Specifically, when stimulated with SARS-CoV-2 peptides *in vitro*, peripheral blood mononuclear cell (PBMC) CD4^+^ and CD8^+^ T cells from individuals aged ≥61 showed a diminished capacity to produce IFN-γ and IL-1β. Although they did not have severe disease, aged individuals also showed a higher frequency of PD-1^+^ cells and significantly diminished IFN-γ/PD-1 ratios among T lymphocytes upon SARS-CoV-2 peptide stimulation. Impaired T cell IL-1β expression coincided with reduced NLRP3 levels in T lymphocytes. However, the expression of these molecules was not affected in the monocytes of individuals aged ≥61. Together, these data reveal SARS-CoV-2-specific CD4^+^ and CD8^+^ T-cell intrinsic cytokine alterations in the individuals older than 61 and may provide new insights into dysregulated COVID-directed immune responses in the elderly.

## Introduction

COVID-19 has continued to spread throughout the world after its initial outbreak in 2019 (1). The primary sites of infection by the SARS-CoV-2 virus are the epithelial cells of the upper respiratory tract and lungs. SARS-CoV-2 infection in the upper respiratory tract is associated with viral transmission and mild symptoms, whereas an infection in the lungs is associated with severe disease. Lymphopenia and reduced cytokine responses in T cells commonly correlate with the severity of SARS-CoV-2 infection and disease, showing the crucial protective role that these T cells play (2, 3). Studies have shown that infected individuals develop SARS-CoV-2-specific T cells that are predominantly associated with T helper 1 (Th1) and T follicular helper (T_FH_) CD4^+^ T cell expansion, along with CD8^+^ T cell responses (4–7). COVID-19 patients with mild disease have demonstrated early induction of interferon-𝛾 (IFN-γ) secreting SARS-CoV-2 specific T cells (8). Animal studies have also confirmed the critical involvement of CD8^+^ and CD4^+^-specific T cells against SARS-CoV-2 in clearing the virus, restricting the severity of the disease, and driving the disease recovery after infection (9–11). Neutralization of IFN-γ is linked to a considerable decrease in survivability post-viral challenge (12). In humans, a slower decline in the viral load combined with immunological misfiring attributed to elevated IFN-α, IFN-γ, and TNF-α (also known as “cytokine storm”) are the characteristic features of severe clinical outcomes (4, 13). The SARS-CoV-2 virion is made up of the spike (S; SPIKE), nucleocapsid (N), and envelope (E) proteins. The S protein facilitates the attachment and entry of this virion via the host cell-surface obligate receptor angiotensin converting enzyme 2 (ACE2) (14). By utilizing the published SARS-CoV-2 sequence and peptide pools, the T cell response against the entire viral proteome of SARS-CoV-2 has been established (15–17). T cells derived from PBMCs are mapped against the 142 T cell epitopes within the SARS-CoV-2 genome, and the dominant epitopes for both CD4^+^ and CD8^+^ T cells come from S, N, and E proteins. SARS-CoV-2 reactive T cells can exist in about 20-60% of individuals who were not previously exposed to SARS-CoV-2 (18) and are likely memory cells that were cross-reactive to other human coronaviruses (15). The presence of these pre-existing SARS-CoV-2-cross-reactive memory T cells may contribute to a lower severity of COVID-19 clinical symptoms and is an area of debate and future research (15, 18–23).

Human aging is associated with systemic inflammation and mucosal manifestations associated with immune dysfunction (24). However, the underlying defects in immune mechanisms are unclear. Age-related immune inflammaging and immunosenescence, and persistent genetic alterations are known to cause a decline in immune functions that protect against infections from pathogens such as SARS-CoV-2 (25, 26). Aging-related immune system malfunction not only reduces the number of naïve CD4^+^ and CD8^+^ T cells but also leads to changes in CD4^+^ and CD8^+^ T cells and CD4+FOXP3+ regulatory T cells (T_regs_) that limit T cell abilities to mount a robust immune response against newer epitopes (27). Aging also increases the number of terminally differentiated CD8^+^ T cells and proliferative senescent T cells, directly contributing to their pro-inflammatory behavior (28). Furthermore, the expression of the co-stimulatory molecule CD28 also declines as CD57 expression increases, resulting in non-proliferative T cells (29). An impaired anti-inflammatory response accompanying old age could also correlate with increased pro-inflammatory immune responses in elderly populations (30). Our results here show reduced expression of IFN-γ, IL-1β, and NLRP3 without changes in activation status in SPIKE-specific CD4^+^ and CD8^+^ T cells in aged COVID+ patients.

## Materials and methods

### COVID+ patients and PBMC isolation

The COVID+ samples were obtained from the University Hospitals biorepository unit, and the study protocol was approved by the Institutional Review Board (University Hospitals, Cleveland). The current study consists of 36 subjects who tested positive for SARS-CoV-2 RNA, as confirmed by Reverse Transcriptase (RT-PCR) of the nasopharyngeal swabs. The samples were collected between May 28, 2020 and Oct 11, 2021, when the major strains in circulation were the original Wuhan-Hu-1 strain, alpha variant (B.1.1.7), and the Delta variant (B.1.617.2). Blood samples were collected from these subjects 2-7 days after the confirmed RT-PCR COVID+ result. Samples from 18 healthy subjects were used as controls. For PBMC isolation, whole blood was collected in EDTA tubes and diluted with sterile phosphate-buffered saline (PBS; 1:1 ratio). The diluted blood was carefully layered over the Ficoll^®^ Paque Plus (GE17-1440-02; 1:1) and centrifuged at 400 ×*g* for 20 min at room temperature with no brake. The PBMC interface was collected and washed twice with PBS-EDTA at 400 ×*g* for 10 min at 4 °C. The PBMC pellets were suspended in a freezing medium (90% fetal calf serum and 10% DMSO) and were cryopreserved in liquid nitrogen until further use. Frozen PBMCs from pre-COVID (before December 2019) uninfected healthy individuals with the exception of 3 samples were used as controls.

### Antibodies and reagents

Fluorochrome conjugated antibodies CD3-BV421 (HIT3a; 740073), CD25-BV786 (M-A251; 563701), PD-1-BV711 (EH12.1; 564017), PD-L1-BV605 (MIH1; 740426), CD38-BV786 (HIT2; 563964), CD14-FITC (M5E2; 557153), CD19-BV711 (SJ25C1; 563036), ROR𝛾t-AF488 (Q21-559; 563621) and Bcl-2-BV450 (Bcl-2/100; 560637) were from BD Biosciences (PA, US). CD4-AF700 (OKT-4; 56-0048-82), FOXP3-PE-Cyanine7 (236A/E7; 25-4777-42), CD3-FITC (HIT3a; 11-039-42), IL-1β-PE (CRM56; 12-7018-82), and IFN-γ-APC (4S-B3; 17-7319-82) were from Thermo Fisher Scientific (CA, US). T-bet- PerCP-Cyanine5.5 (eBio4B10; 45-5825-82) and LAG3- PerCP-eFluor™ 710 (3DS223H; 46-2239-42) are from eBiosciences (CA, US). NLRP3-PE (bs-10021R-PE) is from Bioss (MN, US). TCR stimulating antibodies used in this study were for activating CD3 (HIT3a; 555336;BD biosciences; PA, US) and CD28 (CD28.2; 16-0289-85; Life Technologies corporation; CA, US). Recombinant human IL-1β (C600124-0010) and IL-2 cytokines were from BioBasic Inc. (NY, US). Anakinra (Kineret from Amgen) was a kind gift from Dr. Su at NIAID, NIH.

### SPIKE peptide assay

The cryopreserved PBMCs from healthy controls and COVID+ subjects were rapidly thawed, washed twice in pre-warmed complete RPMI-1640 medium (Cytiva; SH30027FS, supplemented with 10% heat-inactivated human serum, 100 U/ml penicillin, 100 µg/ml streptomycin, 2 mM glutamine and 20 mM HEPES), and suspended in 500 μl pre-warmed complete medium. The viability was assessed using trypan blue stain (BioBasic Inc. NY, US) and flow cytometry-based Live/dead staining (L34968, Thermo Fisher Scientific, CA, US). 1/5^th^ of the total cells were used for *ex vivo* staining or polyclonal stimulation with PMA and ionomycin (PMA-50 ng/ml and ionomycin-500 ng/ml) for 4 h followed by the addition of Brefeldin-A (10 μg/ml) in the last 2 h. The remaining cells were stimulated with PepTivator^®^ SARS-CoV-2 Prot_S Complete (130-127-953, Miltenyi Biotec), which is a pool of lyophilized peptides covering the complete protein coding sequence of the SPIKE glycoprotein of SARS-CoV-2 and capable of stimulating T cells. PBMCs were stimulated with these peptides (1 μg/ml) in the presence of IL-2 (50 U/ml) for 12 h followed by the addition of Brefeldin-A (10 μg/ml) in the last 2 h. Following stimulation, the cells were harvested, fixed and used for intracellular flow cytometry.

### *In vitro* T cell activation

PBMCs from healthy control subjects were stimulated with T cell receptor (TCR) agonists using α-CD3 and α-CD28 antibodies (each 1 μg/ml) in the presence of IL-1β (10 ng/ml) and with or without Anakinra (10 μg/ml). Anakinra was added 10 min before the addition of IL-1β to block the IL-1β receptor. The culture was maintained for 5 days and before the final harvest cells were re-stimulated with α-CD3 antibody(2 μg/ml for 4 h) with the addition of Brefeldin-A (10 μg/ml) in the last 2 h before flow cytometry.

### Flow cytometry

The cells were harvested from their respective conditions after stimulation (either PMA and ionomycin or SPIKE) and pelleted down at 400 ×*g* for 10 min at 4 °C. The cells were segregated for staining panels along with their respective unstained single staining controls and FMO controls. Initially, surface receptors were stained for 1 h at 4 °C with fluorochrome-conjugated antibodies in PBS/BSA followed by Live/dead viability staining using the LIVE/DEAD™ fixable yellow dead cell staining kit (L34968, Thermo Fisher Scientific) for 30 minutes at 4 °C. For intracellular cytokine staining, the cells were fixed using Foxp3/transcription factor fixation/permeabilization buffer set (00-5521-00, Invitrogen) for 45 min at 4 °C. These fixed cells were stained for intracellular cytokines, proteins, and transcription factors using fluorochrome-conjugated antibodies at 4 °C for overnight. Fluorochrome-conjugated antibodies used for intracellular and surface staining were used at 1:50-1:200 dilutions respectively, or according to the manufacturer’s recommended dilution. Flow cytometric acquisition was carried out using BD Fortessa cytometer (BD FACSDiva software ver.7), and for analysis FlowJo 9.8 − 10.7.1 software was used. For examining T cells, we have gated on the live CD3^+^ cells. For examining monocytes, we have gated only on the live CD14^+^CD3^-^cells. Gating strategy included prior gating on viable singlet lymphocytes (**Figure S2A**, gating strategy).

### Statistical analysis

Statistical analysis was performed using GraphPad (Version 8, San Diego, CA). P-values were calculated by Mann-Whitney test assuming random distribution in patient cellular parameter analysis. T test analyses were also used for other *in vitro* experiment analysis. For correlation (r), spearman analysis was used. P < 0.05* was considered significant.

## Results

### CD4/CD8 ratios, frequency of CD4^+^ T_regs_, and PD-1 expression were not altered during acute infection in the blood of COVID+ patients *ex vivo*

**Figure 1:**
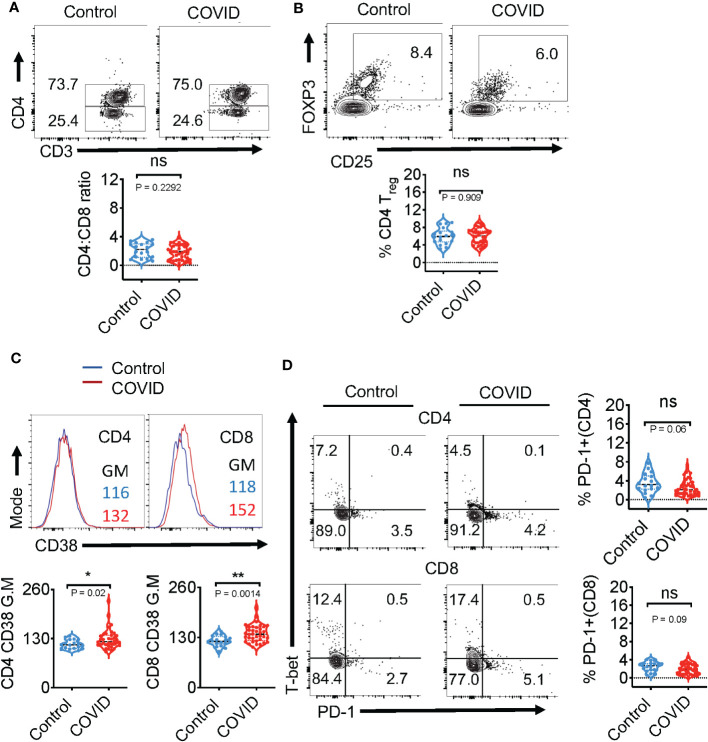
Immunophenotyping of the blood during acute COVID infection shows no changes in CD4/CD8 ratios, frequency of CD4^+^ T_regs_, and PD-1 expression *ex vivo.* Expression of immune cell markers was determined by flow cytometry in healthy controls (n=18) and COVID+ patients with acute SARS-CoV-2 infection (n=36) as described in methods. **(A)** Flow cytometry analysis was conducted to assess CD3^+^CD4^+^ (CD4^+^) and CD3^+^CD4^-^(CD8^+^) T cell ratios. Contour plots (above) and statistical analyses (below) are shown. **(B)** Contour plots (above) and statistical analysis (below) of CD4^+^CD25^+^FOXP3^+^T_regs_ **(C)** Histogram plots of CD4 (left) and CD8 (right) CD38 expression (above) and statistical analysis (below) **(D)** Contour plots show PD-1 (x-axis) and T-bet (y-axis) of CD4 (above) and CD8 (below) and the statistical analysis of PD-1^+^ cells (right). Median values ± SEM are plotted. (Mann-Whitney U test; Two-tailed). *P<0.05, **<0.005. NS, non significant.

We obtained PBMC samples from healthy controls (n=18) and COVID+ patients with acute SARS-CoV-2 infection (n=36) to conduct T cell immunophenotyping *ex vivo* (See **Table 1** for COVID+ patient characteristics). With an exception of two patients, all COVID+ patients presented with mild symptoms (with or without oxygen therapy). All healthy controls with an exception of 3 samples, were collected from the pre-COVID period (before December 2019). Although the overall CD3^+^ T cell proportions were slightly lower in COVID+ patients (data not shown), there were no significant differences in CD4^+^ and CD8^+^ frequencies when compared to uninfected healthy controls (**Figure 1A**). However, the percentages of CD4^+^ and CD8^+^ T cells were diminished in the two COVID+ patients with severe disease who were eventually deceased (data not shown). These T cell trends in COVID+ patients with severe disease were consistent with a previous report, showing that severe lymphopenia correlated strongly with worse COVID severity (31). Also, CD4^+^CD25^+^FOXP3^+^regulatory T cell (T_reg_) percentages and T cell activation marker CD25 were comparable between healthy controls and COVID+ patients (**Figures 1B**, **S1**). However, T cell CD38 expression was moderately and significantly higher in COVID+ patients compared to the healthy controls *ex vivo* (**Figure 1C**). Although PD-1 is a known marker of chronically activated and exhausted T cells (32), it is also a protective memory CD8 T cell marker in SPIKE specific cells in the context of SARS-CoV-2 infection (33). PD-1 is also implicated in T cell dysregulation in COVID+ patients (34). Therefore, we examined the expression of PD-1 along with Th-1 transcription factor T-bet.

**Table 1:** COVID-19 patient characteristics. Primary outcome of the patients was defined by WHO disease severity scale from 1-8. 3 = Hospitalized with mild disease and without oxygen therapy through mask or nasal prongs; 4 = Hospitalized with mild disease and with oxygen therapy through mask or nasal prongs; 8 = Dead.

**Total participants**	**n = 38**
**Total samples included in analysis**	n = 36
**Excluded samples**	2
**Age (years; mean ± SD)**	53.4 ± 12.4
Gender (# and % of participants)	
**Male**	19 (52.7%)
**Female**	17 (47.3%)
**Aged 61 and above (# of participants)**	16
** Male**	7
** Female**	9
**Young 60 and below (# of participants)**	20
** Male**	12
** Female**	8
**Aged- age range**	61 – 70
** Median**	64.24
**Young- age range**	27 – 60
** Median**	45.55
**Aged- severity range**	0 - 8
** Median**	4
**Young- severity range**	0 - 4
** Median**	3
**Aged-Number of patients with severe disease (Deceased)**	2
**Young-Number of patients with severe disease (Deceased)**	0
**Inclusion criteria**	1. Samples and data are contained within the UH Institutional COVID-19 and Coronavirus Biorepository 2. Age range: 18-89 3. Must be tested positive for SARS-CoV-2 RNA, as confirmed through RT-PCR of the nasopharyngeal swabs
**Exclusion criteria**	Any sample or data not obtained in the UH Institutional COVID-19 and Coronavirus Biorepository
**Non-hospitalized/Ambulatory (%)**	30.5
**Hospitalization (%)**	69.4
For hospitalized-WHO disease severity scale*	3, 4, and 8
**Hospitalized without oxygen therapy (%)**	32
**Hospitalized with oxygen therapy (%)**	60
Length of stay (LOS)-Hospitalization	
**(days; mean ± SD)**	7.6 ± 6.6
**LOS (%; < 7 days)**	56
**LOS (%; > 7 days)**	44

When compared to healthy controls, COVID+ patients did not have significant changes in the expression of T-bet (**Figure 1D**-y-axis) and PD-1 (**Figure 1D**-x-axis; right column). These results show that except for the overall activation status, the key phenotypic features of T cells in the PBMCs of COVID+ patients were comparable to those in uninfected healthy controls *ex vivo*.

### SPIKE-specific T cell-IFN-γ is reduced and PD-1 is upregulated in COVID+ patients aged ≥61 *in vitro*

**Figure 2:**
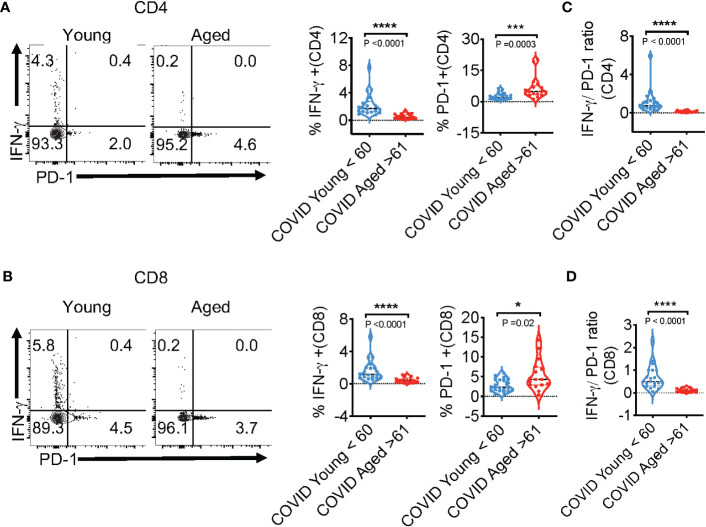
SPIKE-specific T cell-IFN-γ is reduced, but PD-1 is upregulated in COVID+ patients aged ≥61 *in vitro*. COVID-specific responses were assessed in PBMC T cells, comparing COVID+ patients of ages ≤ 60 (n=20; young) and ages ≥61(n=16; aged), by stimulating PBMC with SPIKE peptides *in vitro* for 12 h. **(A, B)** Representative contour plots (left) and statistics (right) showing % of IFN-γ and PD-1**-**expressing CD4^+^ (above) and CD8^+^ (below) cells. % IFN-γ/%PD-1 ratios in CD4^+^ cells **(C)**, and CD8^+^ cells **(D)** *in vitro*. Median values ± SEM are plotted. (Mann-Whitney U test; Two-tailed). *P<0.05, ***<0.0005, ****<0.00005.

We also examined COVID-specific responses in PBMC T cells, comparing COVID**+** patients of age ≤ 60 (n=20; young) and age ≥61 (n=16; aged). SARS-CoV-2-specific T cells were identified from PBMCs after stimulation with peptide pools against the SARS-CoV-2 SPIKE protein to activate both CD8^+^ and CD4^+^-specific T cells for 12 h. The overall viability and CD4:CD8 ratios were comparable between aged and young T cells *ex vivo* (**Figure S2A****;** gating strategy, **Figure S2B****)**. SARS-CoV-2 infection initiates inflammatory cytokine production, leading to a cytokine storm in COVID+ patients. Most patients with severe COVID-19 show higher levels of serum pro-inflammatory cytokines such as IFN-α, IFN-γ, IL-6, IL-12, and TNF-α (35, 36). The preliminary initiation of a cytokine storm is not fully understood, although a large titer of viral antigens discharged by dying cells detected by the immune system is involved. IFNs are cytokines well-known for their antiviral immune responses (37). As such, we measured the T cell anti-viral cardinal cytokine IFN-γ and the immunosenescent/exhaustion marker PD-1 in response to peptide stimulation *in vitro*. While the expression of these proteins was comparable in PBMCs irrespective of age *ex vivo* (**Figure S2C**-y axis, **Figure 1D**), there was significantly blunted expression of SPIKE-specific IFN-γ in aged individuals CD4^+^ and CD8^+^ T cells (**Figures 2A, B**; y axis). As expected, healthy controls naive to COVID-19 also had lower expression of T cell IFN-γ in response to SPIKE peptides *in vitro* (**Figure S3****).** On the other hand, measurement of PD-1 in COVID+ patients showed that aged participant PBMCs contained higher proportions of PD-1-expressing cells in response to SPIKE as compared to subjects age ≤ 60 (**Figures 2A, B**; x axis). However, upon polyclonal stimulation with PMA and ionomycin, age dependent differences in PD-1 and IFN-γ were not observable in COVID+ patients indicating that T cells from individuals of age ≥61 were not generically exhausted (**Figure S4**). Although the expression of PD-1 was slightly higher in PBMCs in T cells from subjects aged ≥61 *ex vivo*, the differences were not statistically significant (**Figure S5**). Remarkably, upon stimulation with SPIKE, we observed a significant reduction of the % IFN-γ/%PD-1 ratio in aged patients CD4 and CD8 compartments (**Figures 2C, D****).** These results demonstrate alterations in SPIKE-specific T cell responses in COVID+ individuals aged ≥61.

### SPIKE-specific T cell- IL-1β expression is reduced in COVID+ patients ages ≥61

**Figure 3:**
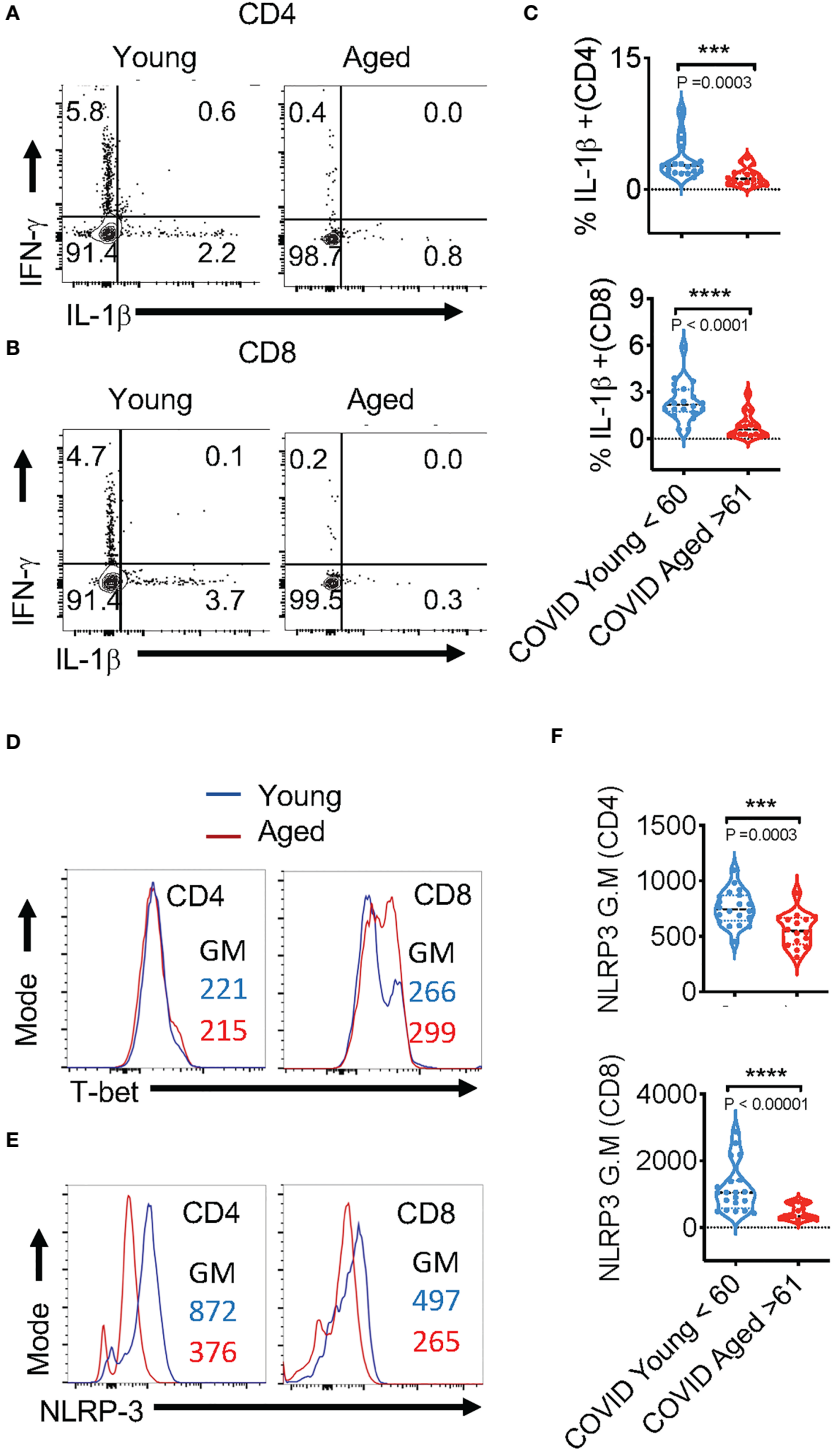
SPIKE-specific T cell-IL-1β expression is reduced in COVID+ patients aged ≥ 61. PBMCs from age ≤ 60 (n=20) and aged ≥ 61 (n=16) COVID+ patients were stimulated with SPIKE peptides in vitro for 12 h to assess COVID-specific responses in T cells. Representative contour plots showing % of PD-1 and IFN-γ expressing **(A, B)**, T-bet **(D)**, NLRP3 **(E)**, of CD4+ (above) and CD8+ (below) cells. Statistics showing % IFN-γ/ %PD-1 ratios **(C)** and NLRP3 expression **(F)**, in CD4+ (top) and CD8+ cells (bottom) in PBMCs from subjects aged ≤ 60 and ≥ 61 in vitro. Median values ± SEM are plotted. (Mann-Whitney U test; Two-tailed). ***<0.0005, ****<0.00005.

IL-1β, a cytokine known for promoting host-protective anti-viral responses has emerged to be a critical cytokine that is upregulated during SARS-CoV-2 infection (38). However, the role of this cytokine on COVID severity has been contentious. Patients with severe or critical COVID-19 infection have considerably higher levels of IL-1β in lung macrophages and bronchoalveolar lavage fluid (BALF) than patients with mild COVID-19 infection (39). Mechanistically, excessive IL-1β can act as a possible risk factor in damaging the hematopoietic stem cells (40). Also, elevated levels of NLRP3 inflammasome activation that serves as a platform for mature IL-1β release is directly related to hyperinflammation as well as increased disease severity of COVID-19 patients (13, 38, 41, 42). However, three other studies have shown that increased IL-1β levels in PBMCs, serum, or plasma did not correlate with COVID-19 severity (13, 42, 43). Furthermore, IL-1β is rather reduced in elderly populations and maybe a risk factor in infections (44–46). Consistently, our previous results also show dysregulated IL-1β/MyD88 signaling and lower levels of salivary IL-1β in individuals aged ≥61 (44). However, this cytokine has not been studied in the context of age-related changes in COVID infection responses. Here we found that the frequency of IL-1β^+^ cells was significantly lower in COVID+ patients aged ≥ 61 in CD4^+^ (**Figures 3A**- x axis, **3C**) and CD8^+^ (**Figures 3B**-x axis, **3C**) populations, when compared to patients aged ≤ 60 in peptide-stimulated PBMCs. While T cells in COVID+ PBMCs had higher levels of cytokine-positive cells compared to uninfected healthy controls (**Figures S3**, **S6****)**, we did not find significant age-associated differences in PD-1 and cytokine expression in COVID+ patients *ex vivo* (**Figures S2**, **S5****)**. Also, upon stimulation of T cells with SPIKE for 12 h, there was no significant difference between both the groups of PBMCs *in vitro* in the expression of T-bet, the transcription factor inducing IFN-γ expression (**Figure 3D****)**. Remarkably, T lymphocytes from the subjects aged ≥ 61 displayed dampened NLRP3 expression in SPIKE-stimulated T lymphocytes *in vitro* (**Figures 3E, F****)**. These results showed that SPIKE-responsive T-cell NLRP3 and IL-1β expression and lower expression of these proteins in lymphocytes from subjects aged ≥61 could have functional effects *in vivo*.

### IL-1β positively correlates with IFN-γ and NLRP3 expression in SPIKE-activated T cells in COVID+ patients

**Figure 4:**
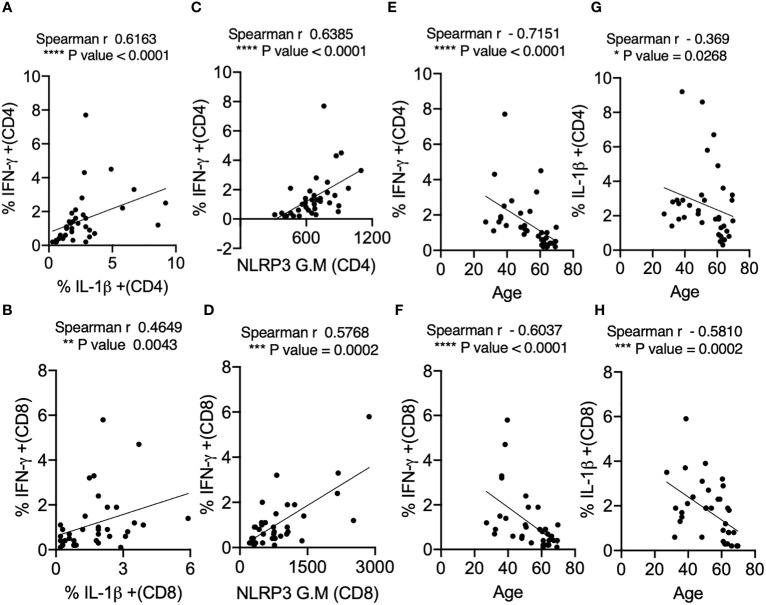
IL-1β expression positively correlates with IFN-γ and NLRP3 expression in SPIKE-activated T cells in COVID+ patients. Spearman Correlation (r) analysis showing positive correlation between IL-1β and IFN-γ in CD4^+^ T cells **(A)** and CD8^+^ T cells **(B)**, and NLRP3 and IFN-γ in CD4^+^ T cells **(C)**, and CD8^+^ T cells **(D)**. Negative correlation between cytokines and age in CD4^+^ T cells **(E, G)** and CD8^+^ T cells **(F, H)**. *P<0.05, **<0.005, ***<0.0005, ****<0.00005.

**Figure 5:**
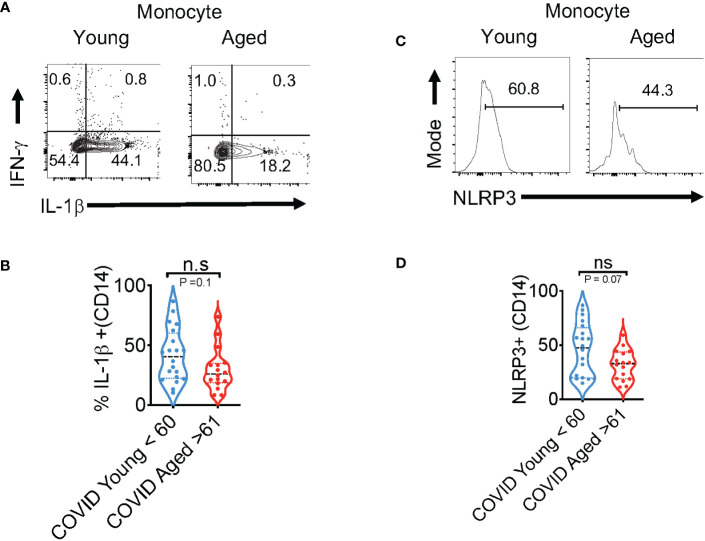
Cytokine dysregulation is not observed in aged monocytes (aged ≥ 61) in SPIKE-activated PBMCs from COVID-infected individuals. PBMCs from COVID+ patients aged ≤ 60 and ≥ 61 were stimulated with SPIKE for 12 h as in **Figure 2**. Representative contour plots **(A)** showing % of IL-1β (x-axis) and IFN-γ (y-axis) gating on CD14^+^ monocytes and statistics of % IL-1β^+^ cells in COVID+ patients aged ≤ 60 and ≥ 61 **(B)**. Histogram plots showing NLRP3 expression **(C)** and statistical analysis of % of NLRP3^+^ cells in COVID+ patients **(D)**. Median values ± SEM are plotted. (Mann-Whitney U test; Two-tailed). NS, non significant.

The *in vivo* importance of the T cell–intrinsic NLRP3 inflammasome in promoting IFN-γ production has been demonstrated (47). This study further showed that IFN-γ production was significantly reduced in NLRP3-deficient CD4^+^ T cells during viral infections. However, T cell intrinsic NLRP3 was not studied. We hypothesized that the NLRP3-IL-1β axis may be involved in regulating T cell IFN-γ expression in COVID+ patients. We determined this correlation between IL-1β and IFN-γ using Spearman analysis and found that antigen-specific IL-1β had a statistically significant positive correlation with IFN-γ expression both in CD4^+^ and CD8^+^ lymphocytes (**Figures 4A, B****)**. Similarly, NLRP3 also correlated with IFN-γ production in both T cell compartments (**Figures 4C, D****)**. Importantly, the frequency of SPIKE-specific IL-1β and IFN-γ T cells showed a significant negative correlation with increased age (**Figures 4E–H**). While the function of the NLRP3-IL-1β axis is more widely implicated in innate cells, and T cell-instructed IL-1β can also activate myeloid cells (48). Therefore, we examined the expression of these proteins in monocytes, gating on CD3^-^CD14^+^ monocyte cells in SPIKE-stimulated PBMCs comparing COVID+ patients aged ≤ 60 and ≥ 61. While monocytes from COVID+ patients expressed higher levels of these cytokines compared to uninfected controls (**Figure S7**, top panel), we observed no significant aging-related differences in IFN-γ and IL-1β expression in COVID+ patients *ex vivo* and during SPIKE stimulation (**Figure S7**, top panel, **Figures 5A, B****)**. Similarly, NLRP3 in monocytes remained unchanged when both the age groups were compared in SPIKE-stimulated PBMCs for 12 h (**Figures 5C, D****)**. Although monocytes expressed higher levels of IFN-γ and IL-1β upon PMA and ionomycin stimulation than in peptide-stimulated cultures, age-related impairment of cytokines was not observed (**Figure S7**, bottom panel). Taken together, these results demonstrated that monocytic IL-1β and NLRP3 were not different between COVID+ patients aged ≤ 60 and ≥ 61 and may not contribute to observed T-cell cytokine changes. These data imply that lower IFN-γ production is directly related to lower COVID-specific T-cell intrinsic NLRP3 and IL-1β expression in SARS-CoV-2-infected individuals aged ≥ 61, and could indicate T cell dysfunction in COVID immune responses independent of monocytic changes with aging.

### IL-1β promotes IFN-γ expression in TCR-activated T cells

**Figure 6:**
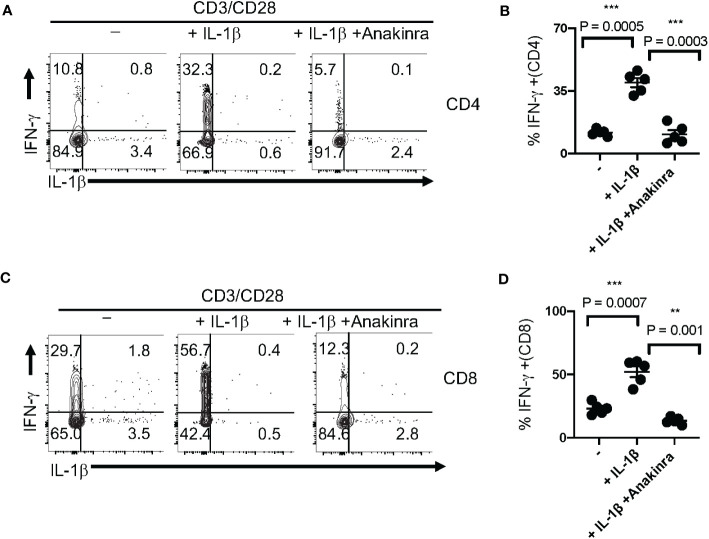
IL-1β promotes IFN-γ expression in T cell receptor (TCR)-activated T cells *in vitro*. **(A)** PBMCs from healthy controls were stimulated with α-CD3 and α-CD28 in the presence of IL-1β (10 ng/ml) with or without Anakinra (10 μg/ml) for 5 days (n=3 independent experiments). Cells were re-stimulated with α-CD3 (2 μg/ml) for 4 h and Brefeldin-A (10 μg/ml) during last 2 h before the flow cytometry assessment. Flow cytometry plots **(A, C)** showing IL-1β (x-axis) and IFN-γ (y-axis) expression (gated on CD3^+^CD4^+^ cells in **B** and gated on CD3^+^CD4^-^ cells in **(D)** and statistical data points showing IFN-γ^+^ T cells from experimental replicates **(B, D)**. Mean values ± SEM from 5 independent experiments are plotted. *P< 0.05; Paired 2-tailed students “t” tests. **<0.005, ***<0.0005.

To determine the functional effect of IL-1β in T cell IFN-γ upregulation, we stimulated T cells with TCR stimulating antibodies α-CD3 and α-CD28 with and without exogenously added IL-1β for 5 days. We found that IL-1β significantly upregulated IFN-γ, which was reversed by Anakinra, an IL-1R antagonist in CD4^+^ (**Figures 6A, B**) and CD8^+^ T cells (**Figures 6C, D**). These data concur with the results from previous studies that showed the effects of IL-1β in antigen-specific T cell activation and *in vivo* (47, 49). Thus, we conclude that impaired IL-1β production may contribute to IFN-γ dysregulation in T cells from subjects aged ≥ 61 during COVID-specific immune responses.

## Discussion

**Figure 7:**
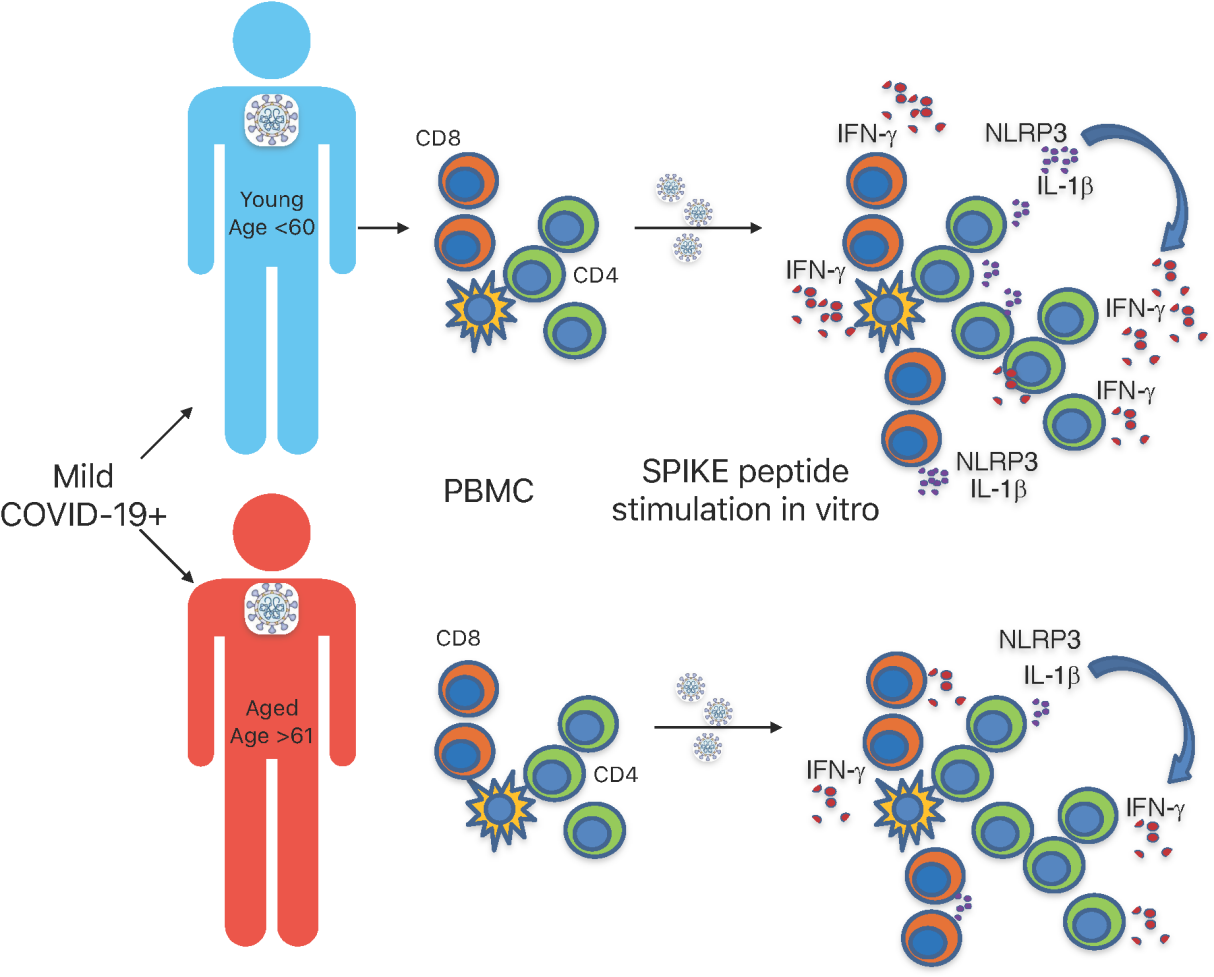
Graphical summary. SPIKE specific T cell defects in IL-1β and IFN-γ response in aged individuals infected with SARS-CoV-2.

Although our study only examined Wuhan strain S-specific responses, and not N or M-specific responses, our results show that T cell-specific defects in IL-1β production may contribute to reduced IFN-γ response in individuals aged ≥ 61 infected with SARS-CoV-2 (**Figure 7**). While the cytokine defects may be related to the higher PD-1 expression in aged COVID+ patients, more work is needed to validate this finding. We initially speculated that the observed results could have been because of T cell exhaustion in individuals aged ≥ 61. However, as shown in **Figure S2**, T cells from these patients, when examined *ex vivo* without the addition of SPIKE peptide, were intrinsically similar to T cells from younger patients in CD4/CD8 ratios and IL-1β and IFN-γ production. We also additionally show that CD38, CD25, T-bet, and PD-1 in these patients (without SPIKE peptide stimulation) *ex vivo*, were comparable in aged and young individuals and they had similar activation and exhaustion statuses (**Figures 1**, **S1**, **3D**, and **S5**). Moreover, when we stimulated these cells through polyclonal stimulation using PMA and ionomycin, the cytokine expression was similar in young and aged patients **Figure S4**, showing that impaired bystander T-cell activation or an exhausted response in aged cells alone did not contribute to lower SPIKE-specific cytokine responses in aged COVID+ patients. Impaired antigen presentation by aged T cells could be one of the mechanisms underlying the observed effects on T cells. With respect to the limited number of cytokines and markers we examined, the monocytes, a potential antigen presenting cells had no cytokine dysregulation (**Figures 5**, **S7**). However, this does not rule out the possibility that antigen presentation might be impaired in monocytes or antigen presenting cells in aged COVID+ patients. While our current study focuses on the effects on T cells, further studies are warranted to study the impact of antigen presentation, co-stimulation and co-inhibition effects on T-cell NLRP3 and cytokine expression. While previous studies have shown changes in T_reg_ frequencies in COVID+ patients, we did not find significant differences between COVID+ patients and uninfected individuals *ex vivo* (**Figure 1B**). However, as shown by CD38 expression, we observed an increased intrinsic activation of T cells in COVID+ patients when compared to healthy controls (**Figure 1C**). We speculate that overall increased T cell activation status irrespective of the age in COVID+ patients could reflect the ongoing acute infection immune response *in vivo*. NLRP3 is induced during SARS-CoV-2 infection (50), but SARS-CoV-2 proteins can over-activate NLRP3 inflammasomes in macrophages and dendritic cells (DC), which are associated with increased disease severity (38, 51–53). Although NLRP3 inflammasome activation and the subsequent inflammation play significant roles in defending against viral infections (54), SARS-CoV-2 N protein-induced NLRP3 inflammasome activation in innate cells is known to cause hyperinflammation (41). We currently do not have data on NLRP3 or IL-1β levels in resident innate cells in the lung or the lung BALF and how they are affected with aging. However, T-cell NLRP3 and IL-1β in patients with severe disease were comparable to those in patients with the mild disease (data not shown), suggesting that T cell intrinsic NLRP3/IL-1β regulation may be distinct from lung-mucosal responses and not directly reflect their COVID severity. There could be potential differences between innate hyperinflammatory NLRP3/IL-1β responses in causing lung pathology and antigen-specific IL-1β responses in blood T cells. In fact, previous reports have shown that increased IL-1β levels in PBMCs, serum, or plasma did not correlate with COVID-19 severity (13, 42, 43). We speculate that while excessive levels of NLRP3/IL-1β are pathogenic, sub-optimal levels may also be a risk factor for secondary infections in elderly population, as shown previously for other infections (44–46). Further studies are warranted to validate this tenet with bigger sample size.

Several studies have demonstrated the association of dysregulated or lower IFN-γ production with severe illness in COVID-19 patients (55–57). Given that, with an exception of two, the COVID+ patients in our cohort had mild infections, the physiological relevance of lower cytokine production in T cells from patients aged ≥ 61, is unclear. While it can be argued that changes in cytokines could be attributed to intrinsic differences in the disease severity within the groups, we did not find any correlation between cytokine levels, length of the stay at the hospital, or presence or absence of oxygen therapy. Stratification of the groups based on length of stay or oxygen therapy was not possible as the group sizes were too small and underpowered. However, stratification of the groups based on the age showed significant differences in the cytokines, which provided the rationale for the study. Importantly the cytokine levels in SPIKE-stimulated T cells showed a significant negative correlation with age (**Figures 4E–H**). A previous report has shown that mild COVID cases are characterized by the presence of a highly clonal population of CD8^+^ T cells in BALF (39). It is tempting to speculate that PBMCs may harbor a proportion of these clonally expanded cells that emigrated from BALF to blood during acute infection. While excessive levels of inflammasome signaling and IL-1β in BALF have been correlated with severity of COVID-19, lower levels of cytokine-expressing T cells in response to SPIKE peptide in aged COVID+ patients with mild disease may be a result of reduced clonal expansion or a reduced frequency of clonally expanded effector or memory cells in these patients *in vivo*. To validate this possibility, more work is warranted to study age-dependent effects on secondary T cell responses comparing BALF and PBMCs in mild versus severe COVID+ patients. Our results are consistent with previous studies showing the effects of IL-1β on antigen-specific IFN-γ responses and T cell-intrinsic IL-1R signaling licensing effector cytokine production in memory CD4^+^ T cells (47, 49, 58), which seem to be impaired during COVID responses in individuals aged ≥61. While we did not see age-associated changes in PBMC monocytes, activated memory T cell-derived IL-1 is known to be involved in T cell sensitization, and DC-mediated production of TNF-α, resulting in local inflammation upon secondary stimulation (58, 59). However, we did not examine the cytokine expression in PBMC DCs, and this remains to be investigated in the future. DCs are known to release IFNs to activate the antiviral cascade in neighboring cells, induce inflammatory cytokine production, recruit inflammatory cells to the infection site, and play a crucial role in restricting SARS-CoV-2 replication. Patients with mild to moderate SARS-CoV-2 demonstrated higher type 1 IFN in the lungs and peripheral blood while patients with comorbidities and elderly patients with extremely high viral loads demonstrated lower levels of type 1 IFN and increased inflammation (55–57). However, the roles of IL-1β and IFN-γ in memory T cells type 1 IFN pathway sensitization and their impairment in aging during the acute or secondary SARS-CoV-2 infection remain to be elucidated. Taken together, our study has revealed a previously unknown impairment of COVID-specific T cell cytokines that could be involved in altered memory responses in individuals aged ≥61. Such an understanding of age-related T cell alterations will contribute to devising therapeutic treatments and improved vaccination strategies specific for the vulnerable elderly population.

## Data availability statement

The original contributions presented in the study are included in the article/**Supplementary Material**. Further inquiries can be directed to the corresponding author.

## Ethics statement

The studies involving humans were approved by University Hospitals Institution review board. The studies were conducted in accordance with the local legislation and institutional requirements. The participants provided their written informed consent to participate in this study.

## Author contributions

PP conceptualized the study, designed and performed experiments, analyzed data, and supervised the project. SM performed the stimulation and staining experiments and contributed to manuscript writing. HD provided the optimal stimulation peptides for cell cultures. SJ performed some cell-culture experiments and provided technical assistance with patient sample procurement, processing, paperwork, and assessing the data in a masked fashion. AA contributed to writing. DA, JJ, and CS contributed to concept and scientific discussions and critically read the manuscript. All authors contributed to the article and approved the submitted version.
