# Supplementary material for: Distinct SARS-CoV-2 specific NLRP3 and IL-1β responses in T cells of aging patients during acute COVID-19 infection
Source: Front Immunol. 2023 Sep 18;14:1231087. doi: 10.3389/fimmu.2023.1231087 (PMC10548880; doi:10.3389/fimmu.2023.1231087)
Supplement: Supplementary file 1 [file DataSheet_1.pdf]

Supplementary figures

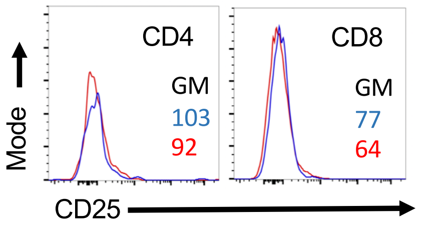

Fig.S1. CD25 expression *ex vivo.* CD25 expression was determined by flow cytometry in healthy controls (n=18) and COVID patients with acute SARS-CoV-2 infection (n=36) as described in methods. Flow cytometry analysis was conducted to assess CD3+CD4+ (CD4) and CD3+CD4-(CD8+) T cell ratios. Histogram plots are shown.

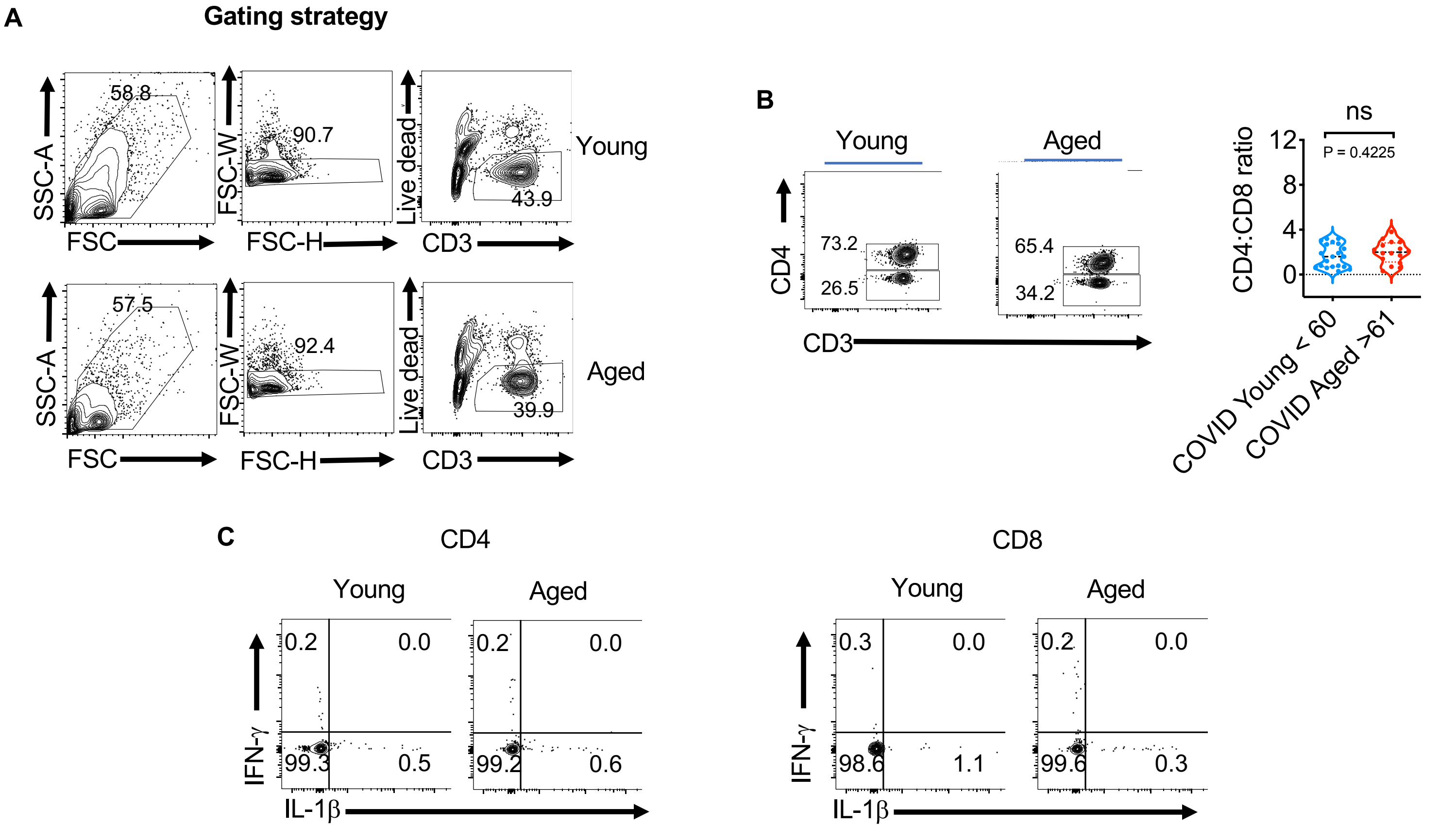

Fig.S2. A) Gating strategy. Lymphocyte gating (FSC-A vs SSC-A), singlet gating (FSC-H vs FSC-W), and viable CD3 gating to exclude dead cells (CD3 vs live dead stain) were employed in all flow cytometric analysis of T cells. Aged versus young cells are compared here. **B**) **CD4/CD8 ratio in aged and young cells**. PBMC were gated as in A, and CD4 and CD8 proportions were determined in CD3+ T cells. Contour plots (left) and statistical analysis (right) show the CD4/CD8 ratio. **C**) **IL-1β and IFN-γ expression in young vs aged *ex vivo.*** Cytokine expression was assessed in T cells of PBMC, comparing young (n=20) and aged (n=16) COVID patients *ex vivo*. Representative contour plots showing cytokine expressing CD4+ (left) and CD8+ (right) cells.

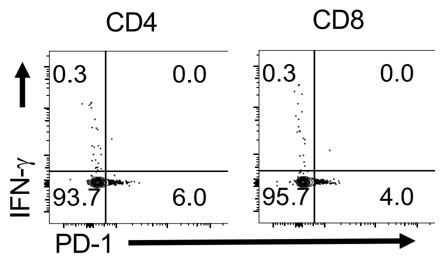

Fig.S3.PD-1 and IFN-γ expression expression in healthy controls*.* Representative contour plots showing the expression in CD4+ (left) and CD8+ (right) cells in PBMC stimulated with SPIKE peptides *in vitro.*

Supplementary figures

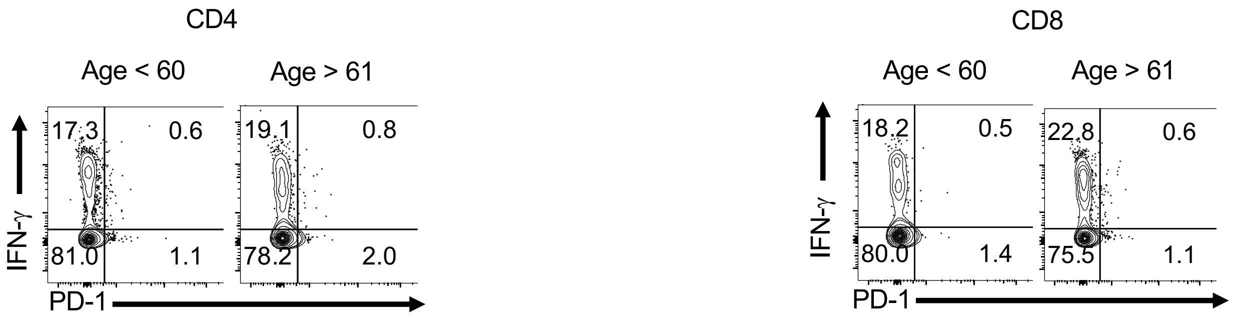

Fig.S4. PD-1 and IFN-γ expression in T cells upon polyclonal stimulation of aged versus young COVID patients. Representative contour plots showing the cytokine expression in CD4+ (left) and CD8+ (right) cells in PBMC stimulated with PMA/Ionomycin for 4 h *in vitro.*

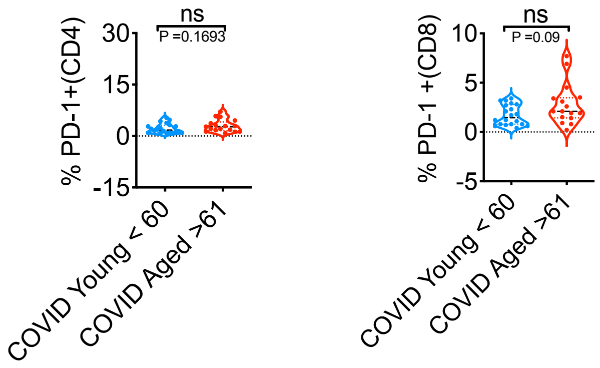

Fig.S5. PD-1 expression in young vs aged *ex vivo.* PD-1 expression was assessed in T cells of PBMC, comparing young (n=20) and aged (n=16) COVID patients *ex vivo*. Statistical analysis showing % PD-1 expressing CD4+ (left) and CD8+ (right) cells.

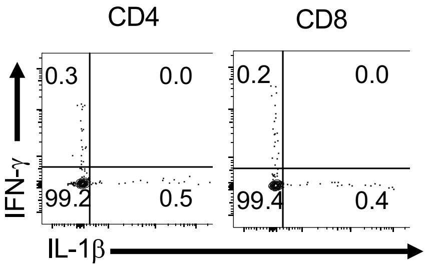

Fig.S6. IL-1β and IFN-γ expression in healthy controls. Representative contour plots showing the cytokine expression in CD4+ (left) and CD8+ (right) cells in PBMC stimulated with SPIKE peptides *in vitro.*

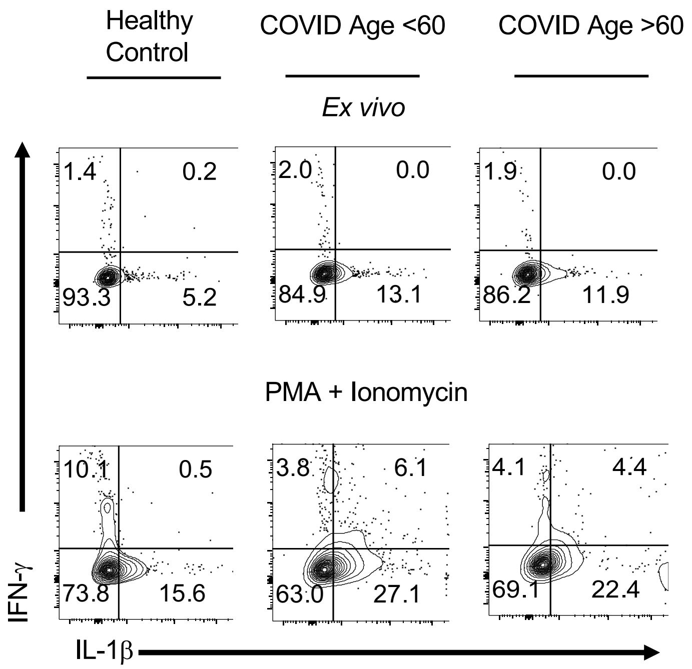

Fig.S7. IL-1β and IFN-γ expression in monocytes in PBMC from healthy controls and COVID-infected individuals. PBMC from aging and young COVID+ patients *ex vivo* or were stimulated with PMA and ionomycin as in Fig.2. Representative contour plots showing % of IL-1β (x-axis) and IFN-γ (y-axis) gating on CD14+ monocytes in aged vs young COVID+ patients.
